# Type-specific effects of orofacial pain on sleep quality: a cross-sectional clinical study

**DOI:** 10.22514/jofph.2026.011

**Published:** 2026-01-12

**Authors:** Sümeyye Coşgun-Baybars, Merve Hacer Talu, Hacer Yalçin, Dicle Gökdemir

**Affiliations:** ^1^Department of Oral and Maxillofacial Radiology, Fırat University, 23119 Elazığ, Türkiye; ^2^Department of Oral and Maxillofacial Radiology, Istanbul Nişantaşı University, 34398 Istanbul, Türkiye; ^3^Private Practice, 23119 Elazığ, Türkiye

**Keywords:** Orofacial pain, Sleep quality, Pittsburgh sleep quality index, Neuropathic pain, Temporomandibular disorders

## Abstract

**Background**: This study aimed to assess the impact of different types of orofacial pain on sleep quality and to examine the influence of age and gender on sleep-related parameters. **Methods**: In this cross-sectional study, 400 patients with orofacial pain presenting to the Faculty of Dentistry, Fırat University, were included. Participants were divided into eight pain categories: pulpal, periodontal, impacted tooth-related, dental implant-related, temporomandibular disorder-related, mucosal/cutaneous, neuropathic, and oncologic. Pain intensity was measured using the Numeric Rating Scale, and sleep quality was assessed via the Pittsburgh Sleep Quality Index (PSQI). Non-parametric tests and correlation analyses were used for statistical evaluation. **Results**: The mean age was 33.62 ± 13.06 years, and 66.8% were female. The mean global PSQI score was 5.56 ± 2.84. Neuropathic and mucosal/cutaneous pain groups demonstrated significantly higher PSQI scores, especially in sleep latency and disturbances (*p* < 0.05). Females had significantly higher scores in sleep latency, disturbances, and daytime dysfunction than males (*p* < 0.05). Age was weakly but significantly correlated with several PSQI components. **Conclusions**: Neuropathic and mucosal/cutaneous pain types were associated with the most detrimental effects on sleep quality. Gender and age were also found to influence specific sleep parameters.

## 1. Introduction

Orofacial pain may originate from pulpal, periodontal, mucosal, neuropathic, infective, post-surgical events, or pericoronitis, or even temporomandibular disorders (TMD), and it is often influenced not only by sensory stimuli but also by emotional stress and functional impairment, which can directly affect sleep physiology [1, 2]. The relationship between sleep and pain represents one of the few bidirectional physiological interactions identified in contemporary clinical science. Impaired sleep quality has been shown to increase pain sensitivity, while chronic pain negatively impacts sleep duration, quality, and pattern, thereby perpetuating this cycle [3, 4]. The clinical implications of this reciprocal relationship are particularly evident in the context of orofacial pain. Recent studies have demonstrated that conditions such as TMD, neuropathic facial pain, mucosal lesions, and potential bruxism are associated with detrimental effects on sleep duration, latency, and overall quality [5, 6, 7]. Sleep disturbances, in turn, may intensify the symptoms of these conditions, creating a vicious cycle [8, 9]. Furthermore, individual variables, such as gender and age, are known to affect both pain perception and sleep parameters in distinct ways [10, 11]. Especially among women, a higher emotional burden and increased sensitivity to pain may contribute to a more complex interaction between pain and sleep [6].

However, most existing studies in the literature focus on a single type of orofacial pain, and there is a notable lack of systematic and comparative investigations assessing the differential impact of various orofacial pain types on sleep quality [12, 13]. Additionally, sleep problems specific to the orofacial region are often overlooked in the broader medical sleep literature, potentially leading to incomplete treatment planning in clinical practice [7, 14]. In this context, the present cross-sectional observational study aimed to comparatively evaluate the impact of different types of orofacial pain on sleep quality in patients presenting to dental clinics with pain-related complaints. The influence of demographic variables, such as age and gender, on specific components of sleep quality was also analyzed. By systematically examining a broader spectrum of pain types, this study seeks to enrich the current body of clinical knowledge regarding the sleep-pain relationship in orofacial conditions and to support a multidisciplinary approach to patient management.

## 2. Materials and methods

### 2.1 Study design and participants

This study was designed as a cross-sectional observational investigation conducted on patients presenting with orofacial pain complaints to the Department of Oral and Maxillofacial Radiology at the Faculty of Dentistry, Fırat University. This study was initiated under the scope of the 2209-A—Research Project Support Programme for Undergraduate Students, funded by the Scientific and Technological Research Council of Türkiye (TÜBİTAK). Within the scope of the research, the types of orofacial pain experienced by the patients were identified, and their relationship with sleep quality was evaluated. Between March and July 2025, all patients presenting with orofacial pain complaints to the Department of Oral and Maxillofacial Radiology at the Faculty of Dentistry, Fırat University, were screened. The initial evaluation consisted of a standardized medical history and clinical examination conducted by two calibrated oral and maxillofacial radiology specialists. Subsequently, eligibility was assessed according to the inclusion and exclusion criteria. Consecutive patients who met the criteria and provided written informed consent were enrolled in the study. In total, 2400 patients were screened, of whom 400 met the criteria and were included in the analysis.

Ethical approval was obtained from the Non-Interventional Research Ethics Committee of Fırat University (Approval No: 2025/03-18-31994; Date: 13 February 2025), and data were collected thereafter, between March and July 2025.

### 2.2 Inclusion criteria

Age between 12 and 70 years;

Presentation to a healthcare institution within the past month with complaints corresponding to the pain types defined in the study;

A Numeric Rating Scale (NRS) pain score of 4 or higher;

Voluntary participation in the study with signed informed consent.

### 2.3 Exclusion criteria

A prior diagnosis of chronic sleep disorder;

A history of psychiatric illness or current use of psychotropic medications that may affect sleep patterns;

Undergoing any surgical intervention or having a history of acute systemic illness within the past month;

Use of any pharmacological treatment—such as analgesics, non-steroidal anti-inflammatory drugs (NSAIDs), muscle relaxants, or combinations thereof—within 48–72 hours prior to the start of the study;

Receiving any form of physiotherapy, manual therapy, or similar physical intervention within 15 days prior to data collection; and

Presence of orofacial complaints not classified among the pain types defined in the study (*i.e.*, pulpal, periodontal, impacted tooth-related, dental implant-related, TMD-related, mucosal/cutaneous, neuropathic, or oncologic in origin).

### 2.4 Sample size

The study included a total of 400 patients aged between 12 and 64 years, with a mean age of 33.62 ± 13.06 years. Among the participants, 33.3% (n = 133) were male and 66.8% (n = 267) were female.

### 2.5 Data collection methods

The type of orofacial pain experienced by each participant was determined through a detailed medical history and clinical examination. The pain types were categorized as follows:

Pulpal pain was confirmed by thermal (cold) testing, percussion sensitivity, and periapical radiographic findings.

Periodontal pain was identified through periodontal probing, tooth mobility, and radiographic evidence of bone loss.

Impacted tooth-related pain was determined based on clinical examination findings and panoramic/cone-beam computed tomography (CBCT) imaging.

Implant-related pain was assessed using peri-implant probing, radiographic bone loss, and the diagnostic criteria for peri-implant mucositis/peri-implantitis.

TMD-related pain was evaluated according to the Diagnostic Criteria for Temporomandibular Disorders (DC/TMD), including palpation tenderness, range of mandibular motion, and the presence of clicking or crepitation.

Mucosal/cutaneous pain was diagnosed through clinical examination and, when indicated, confirmed by histopathological analysis.

Neuropathic pain was defined according to the International Headache Society diagnostic criteria for trigeminal neuralgia.

Oncologic pain was based on clinical findings in conjunction with histopathologically confirmed malignancy. For easier interpretation by general clinicians, simplified clinical analogies for the eight pain types analyzed in this study are provided in **Supplementary Table 1** (*e.g.*, pulpal pain ≈ root canal pain, periodontal pain ≈ scaling/root planing, oncologic pain ≈ post-radiation pain, *etc.*). Additionally, a detailed medical history was obtained from each patient, documenting the location, duration, course, and frequency of pain. The acute or chronic nature of pain was determined according to symptom duration: pain lasting less than 1 month was classified as acute, while pain persisting for 1 month or longer was classified as chronic.

### 2.6 Pain assessment method

Pain intensity was assessed using the Numeric Rating Scale (NRS), a validated 0–10 scale where 0 indicates no pain and 10 indicates the worst imaginable pain. A threshold score of ≥4 was used to define moderate-to-severe pain, ensuring methodological consistency with the study’s objective [15]. The interpretation of NRS scores was as follows: 0 = No pain; 1–3 = Mild pain; 4–6 = Moderate pain; 7–10 = Severe pain.

### 2.7 Sleep assessment method

The sleep quality of participants was assessed using the PSQI. The PSQI is a validated and reliable self-report questionnaire developed to evaluate overall sleep quality over the past month. It consists of 19 items grouped into seven components that assess various aspects of sleep habits and disturbances:

Subjective Sleep Quality;

Sleep Latency;

Sleep Duration;

Habitual Sleep Efficiency;

Sleep Disturbances;

Use of Sleep Medication; and

Daytime Dysfunction.

Each component is scored on a scale from 0 to 3, and the total global score ranges from 0 to 21. A total PSQI score greater than 5 is considered indicative of “poor sleep quality”. The structure of the index allows for a detailed analysis of both overall sleep quality and specific problem areas related to sleep. The validity and reliability of the PSQI have been confirmed for use in the Turkish population [16].

### 2.8 Statistical analysis

Data obtained in the study were analyzed using IBM SPSS Statistics for Windows, Version 22.0 (IBM Corp., Armonk, NY, USA). The normality of the distribution of parameters was assessed using the Kolmogorov-Smirnov test, which indicated that the data did not follow a normal distribution. Accordingly, the Kruskal-Wallis test was employed for comparisons among multiple groups, and Dunn’s *post hoc* test was used to identify pairwise differences when significant results were observed. The Mann-Whitney U test was applied for comparisons between two independent groups, while the Chi-square test was used for the analysis of categorical variables. Relationships between variables were examined using Spearman’s rho correlation analysis. Descriptive statistics included minimum, maximum, mean, standard deviation, median, and frequency values. A *p*-value of less than 0.05 was considered statistically significant. Subgroup comparisons (*e.g.*, neuropathic pain) were prespecified as exploratory and primarily descriptive. Given the small sample sizes in some strata, the study was not powered for definitive between-group inferences in these subgroups. Findings should therefore be interpreted with caution.

## 3. Results

This study was conducted on a total of 400 individuals aged between 12 and 64 years, with a mean age of 33.62 ± 13.06 years. Among the participants, 133 (33.3%) were male and 267 (66.8%) were female. Of the participants, 28.5% reported pulpal pain, 27% periodontal pain, 16% pain related to impacted teeth, 12% TMD-related pain, 5% dental implant-associated pain, 5% mucosal or cutaneous pain, 3.5% oncologic pain, and 3% neuropathic pain (Fig. 1). This distribution indicates that pulpal and periodontal pain were the most frequently reported types of dental pain, while neuropathic and oncologic pain were comparatively less common. Among the 400 patients included in the study, 68.5% presented with acute pain and 31.5% with chronic pain. Neuropathic and oncologic pain were more frequently observed in the chronic pain group, whereas pulpal and impacted tooth-related pain predominated in the acute pain group. Participants with chronic pain had significantly higher total PSQI scores compared to those with acute pain (*p * < 0.05). Given the small sample size in the neuropathic pain subgroup (n = 12), the estimates carry wide confidence intervals and should be interpreted with caution.

**Fig. 1.** Pie chart illustrating the proportional distribution of eight different orofacial pain types among the 400 individuals included in the study. TMD: temporomandibular disorders.

The total PSQI score ranged from 0 to 15, with a mean of 5.56 ± 2.84 and a median of 5. Descriptive statistics for the PSQI total and subcomponent scores are presented in Table 1. The seven subcomponents—subjective sleep quality, sleep latency, sleep duration, habitual sleep efficiency, sleep disturbances, use of sleep medications, and daytime dysfunction—were scored on a scale of 0 to 3. Among these, sleep disturbances had the highest mean score (1.33 ± 0.59; median = 1), indicating a notable prevalence of sleep irregularities among participants. Similarly, the sleep latency component also yielded a relatively high mean score (1.30 ± 0.93; median = 1), suggesting considerable variability in the time required to fall asleep. These findings demonstrate a heterogeneous distribution of sleep quality and indicate that sleep disturbances were present in a subset of individuals.

**Table 1.** Descriptive statistics of PSQI subcomponents and total score.

PSQI Component	Minimum	Maximum	Mean ± SD	Median (IQR)
Subjective Sleep Quality	0	3	0.67 ± 0.94	0 (0–1)
Sleep Latency	0	3	1.30 ± 0.93	1 (1–2)
Sleep Duration	0	3	0.43 ± 0.82	0 (0–1)
Habitual Sleep Efficiency	0	3	0.22 ± 0.54	0 (0–0)
Sleep Disturbances	0	3	1.33 ± 0.59	1 (1–2)
Use of Sleep Medication	0	3	1.11 ± 0.65	1 (1–2)
Daytime Dysfunction	0	3	0.51 ± 0.71	0 (0–1)
Total PSQI Score	0	15	5.56 ± 2.84	5 (4–7)

*Data are presented as Mean ± Standard Deviation and Median (Interquartile Range). PSQI subcomponent scores range from 0 to 3, and the global score ranges from 0 to 21. PSQI: Pittsburgh Sleep Quality Index; SD: Standard Deviation; IQR: Interquartile Range.*

A statistically significant difference was observed in gender distribution across the pain groups (*p* = 0.001; *p * < 0.05). Oncologic pain was more frequently observed in males (71.4%), whereas neuropathic pain (83.3%), impacted tooth-related pain (78.1%), and pulpal pain (73.7%) were more prevalent among females (Table 2). There was also a statistically significant difference in the mean age between the pain groups (*p* = 0.001). The mean age of individuals with impacted tooth-related pain was significantly lower than that of individuals with periodontal pain (*p* = 0.003), dental implant-associated pain (*p* = 0.001), mucosal and cutaneous pain (*p* = 0.001), neuropathic pain (*p* = 0.001), and oncologic pain (*p* = 0.001). Conversely, individuals with oncologic pain had a significantly higher mean age compared with those with pulpal pain (*p* = 0.001), periodontal pain (*p* = 0.001), impacted tooth-related pain (*p* = 0.001), and TMD-related pain (*p* = 0.001). Likewise, individuals with neuropathic pain had a significantly higher mean age than those with pulpal pain (*p* = 0.021), impacted tooth-related pain (*p* = 0.001), and TMD-related pain (*p* = 0.007). Additionally, the mean age of individuals with oncologic pain was significantly higher than that of individuals with pulpal pain (*p* = 0.004), impacted tooth-related pain (*p* = 0.001), and TMD-related pain (*p* = 0.002). No statistically significant differences in mean age were observed among the other pain groups (*p * > 0.05) (Table 2).

**Table 2.** Gender and age distribution by pain type.

Pain Type	Male n (%)	Female n (%)	Age (yr), Mean ± SD	Median (IQR), yr
Pulpal Pain	30 (26.3)	84 (73.7)	31.75 ± 12.39	31.0 (23–40)
Periodontal Pain	50 (46.3)	58 (53.7)	34.57 ± 12.11	34.0 (26–42)
Impacted tooth-related pain	14 (21.9)	50 (78.1)	26.09 ± 6.61	25.0 (21–30)
Dental implant pain	11 (55.0)	9 (45.0)	43.90 ± 12.82	48.0 (35–53)
TMD-related pain	10 (20.8)	38 (79.2)	30.00 ± 12.41	23.0 (19–38)
Mucosal/cutaneous pain	6 (30.0)	14 (70.0)	39.65 ± 11.59	44.0 (33–48)
Neuropathic pain	2 (16.7)	10 (83.3)	46.17 ± 14.11	45.5 (38–55)
Oncologic pain	10 (71.4)	4 (28.6)	54.14 ± 11.82	60.0 (48–63)
*p*-value	0.001^1^	0.001^2^	0.001^2^	

*Data are presented as n (%) for categorical variables, and Mean ± Standard Deviation and Median (Interquartile Range) for continuous variables. ^1^Chi-square test, ^2^Kruskal-Wallis test. p < 0.05 indicates statistical significance. TMD: temporomandibular disorders; SD: Standard Deviation; IQR: Interquartile Range.*

Mean sleep latency scores differed significantly among the pain groups (*p* = 0.003; *p * < 0.05). Individuals with mucosal and cutaneous pain had significantly higher sleep latency scores compared with those with pulpal (*p* = 0.004), periodontal (*p* = 0.001), impacted tooth-related (*p* = 0.001), dental implant-associated (*p* = 0.003), and TMD-related pain (*p * < 0.05). Similarly, participants with neuropathic pain exhibited significantly higher sleep latency scores compared with those with pulpal (*p* = 0.020), periodontal (*p* = 0.005), impacted tooth-related (*p* = 0.008), dental implant-associated (*p* = 0.010), and TMD-related pain (*p* = 0.006) (*p * < 0.05). No significant differences in sleep latency were observed among the other pain groups (*p * > 0.05).

Mean sleep duration differed significantly across the pain groups (*p* = 0.010; *p * < 0.05). Individuals with neuropathic pain reported significantly longer sleep duration than those with impacted tooth-related pain (*p* = 0.017; *p * < 0.05), while sleep duration did not differ among the other groups (*p * > 0.05).

Sleep disturbance scores also varied significantly between pain groups (*p* = 0.001; *p * < 0.05). Participants with mucosal and cutaneous pain showed higher disturbance scores than those with periodontal (*p* = 0.002), impacted tooth-related (*p* = 0.001), and TMD-related pain (*p * < 0.05). Similarly, individuals with oncologic pain scored higher than those with periodontal (*p* = 0.029), impacted tooth-related (*p* = 0.013), and TMD-related pain (*p* = 0.004) (*p * < 0.05). The remaining groups showed no significant differences in sleep disturbance (*p * > 0.05).

Use of sleep medication also differed significantly across the pain groups (*p* = 0.001; *p * < 0.05). Individuals with mucosal and cutaneous pain reported greater medication use than those with pulpal (*p* = 0.003), periodontal (*p* = 0.004), impacted tooth-related (*p* = 0.001), and dental implant-associated pain (*p* = 0.001). Participants with oncologic pain also used more sleep medication than those with impacted tooth-related (*p* = 0.007) and dental implant-associated pain (*p* = 0.010) (*p * < 0.05). No other significant group differences emerged (*p * > 0.05).

Total PSQI scores also showed significant variation among the pain groups (*p* = 0.004; *p * < 0.05). Individuals with mucosal and cutaneous pain had higher total scores than those with pulpal (*p* = 0.002), periodontal (*p* = 0.003), impacted tooth-related (*p* = 0.001), and dental implant-associated pain (*p* = 0.001) (*p * < 0.05). Other groups did not differ significantly in total PSQI scores (*p * > 0.05). In addition, subjective sleep quality, habitual sleep efficiency, and daytime dysfunction scores did not differ significantly among the pain groups (*p * > 0.05) (Table 3).

**Table 3.** PSQI subcomponents and total score by pain type.

Pain Type	Subjective Sleep Quality	Sleep Latency	Sleep Duration	Habitual Sleep Efficiency	Sleep Disturbances	Use of Sleep Medication	Daytime Dysfunction	Total PSQI Score
Pulpal Pain	0.68 ± 0.89; 0.0 (0–1)	1.32 ± 0.89; 1 (1–2)	0.37 ± 0.79; 0 (0–0)	0.25 ± 0.58; 0 (0–0)	1.35 ± 0.51; 1 (1–2)	1.07 ± 0.56; 1 (1–1)	0.49 ± 0.73; 0 (0–1)	5.54 ± 2.71; 5 (4–7)
Periodontal Pain	0.70 ± 0.90; 0.0 (0–1)	1.20 ± 0.87; 1 (1–2)	0.48 ± 0.96; 0 (0–1)	0.17 ± 0.42; 0 (0–0)	1.30 ± 0.57; 1 (1–2)	1.07 ± 0.69; 1 (1–1)	0.54 ± 0.72; 0 (0–1)	5.46 ± 2.69; 5 (4–7)
Impacted tooth-related pain	0.66 ± 1.06; 0.0 (0–1)	1.19 ± 0.89; 1 (0–2)	0.19 ± 0.47; 0 (0–0)	0.22 ± 0.60; 0 (0–0)	1.22 ± 0.70; 1 (1–2)	0.91 ± 0.64; 1 (0–1)	0.56 ± 0.75; 0 (0–1)	4.94 ± 2.92; 4 (3–6)
Dental implant pain	0.20 ± 0.62; 0.0 (0–0)	1.10 ± 0.72; 1 (0–2)	0.60 ± 0.94; 0 (0–1)	0.00 ± 0.00; 0 (0–0)	1.35 ± 0.49; 1 (1–2)	0.80 ± 0.62; 1 (0–1)	0.40 ± 0.75; 0 (0–1)	4.45 ± 1.54; 4 (3–5)
TMD-related Pain	0.71 ± 0.99; 0.0 (0–1)	1.17 ± 1.04; 1 (0–2)	0.54 ± 0.77; 0 (0–1)	0.27 ± 0.54; 0 (0–0)	1.17 ± 0.63; 1 (1–2)	1.21 ± 0.58; 1 (1–2)	0.46 ± 0.65; 0 (0–1)	5.52 ± 2.61; 5 (4–7)
Mucosal/cutaneous Pain	0.80 ± 1.01; 0.5 (0–1)	2.00 ± 0.79; 2 (2–3)	0.60 ± 1.05; 0 (0–1)	0.30 ± 0.47; 0 (0–1)	1.70 ± 0.47; 2 (1–2)	1.70 ± 0.66; 2 (1–2)	0.80 ± 0.77; 1 (0–1)	7.90 ± 3.45; 6.50 (6–9)
Neuropathic Pain	0.67 ± 0.98; 0.0 (0–1)	2.00 ± 1.04; 2 (1–3)	0.83 ± 0.72; 1 (0–1)	0.00 ± 0.00; 0 (0–0)	1.67 ± 0.49; 2 (1–2)	1.50 ± 0.80; 1 (1–2)	0.33 ± 0.49; 0 (0–1)	7.00 ± 3.07; 7.50 (5–9)
Oncologic Pain	0.71 ± 1.20; 0.0 (0–1)	1.43 ± 1.22; 1 (0–2)	0.29 ± 0.73; 0 (0–0)	0.50 ± 1.09; 0 (0–1)	1.71 ± 0.73; 2 (1–2)	1.57 ± 0.51; 2 (1–2)	0.29 ± 0.47; 0 (0–0)	6.50 ± 3.65; 7 (5–9)
*p*-value	0.368	0.003*	0.010*	0.145	0.001*	0.001*	0.413	0.004*

Data are presented as Mean ± Standard Deviation; Median (Interquartile Range). Kruskal-Wallis test. *p < 0.05 indicates statistical significance. TMD: temporomandibular disorders; PSQI: Pittsburgh Sleep Quality Index.

In the gender-based analysis, females had significantly higher scores than males in subjective sleep quality, sleep latency, sleep disturbances, sleep medication use, daytime dysfunction, and the total PSQI score (all *p * < 0.05), reflecting poorer overall sleep quality. By contrast, no significant differences were found between sexes for sleep duration or habitual sleep efficiency (*p * > 0.05).

In the correlation analysis (Table 4), age showed weak but statistically significant associations with several PSQI subcomponents. Older age was related to lower subjective sleep quality (*r* = −0.103, *p* = 0.040) and higher scores for sleep duration (*r* = 0.218, *p* = 0.001), sleep disturbances (*r* = 0.154, *p* = 0.002), and use of sleep medication (*r* = 0.103, *p* = 0.039). No significant correlations were observed between age and sleep latency, habitual sleep efficiency, daytime dysfunction, or the total PSQI score (all *p * > 0.05).

**Table 4.** Correlations between age and PSQI subcomponents and total score.

PSQI Component	*r*	*p*-value
Subjective Sleep Quality	−0.103	0.040*
Sleep Latency	0.083	0.096
Sleep Duration	0.218	0.001*
Habitual Sleep Efficiency	−0.033	0.508
Sleep Disturbances	0.154	0.002*
Use of Sleep Medication	0.103	0.039*
Daytime Dysfunction	−0.088	0.080
Total PSQI Score	0.052	0.298

Spearman’s rho correlation test. *p < 0.05 indicates statistical significance. PSQI: Pittsburgh Sleep Quality Index.

Among male participants, pain types showed significant differences in several sleep quality parameters (**Supplementary Table 2**). Subjective sleep quality varied across groups (*p* = 0.036); men with oncologic pain scored higher than those with TMD-related pain (*p* = 0.017), mucosal and cutaneous pain (*p* = 0.039), and neuropathic pain (*p* = 0.046), while no other group differences were noted (*p * > 0.05). For sleep latency, pain groups also differed significantly (*p* = 0.046). Male participants with TMD-related pain reported lower latency scores compared with those with pulpal (*p* = 0.030), mucosal/cutaneous (*p* = 0.009), and neuropathic pain (*p* = 0.024). The remaining groups did not differ (*p * > 0.05). Sleep disturbance scores varied markedly across pain types (*p* = 0.001); men with mucosal/cutaneous pain scored higher than those with pulpal (*p* = 0.001), periodontal (*p* = 0.001), impacted tooth-related (*p* = 0.001), dental implant-associated (*p* = 0.017), TMD-related (*p* = 0.001), and neuropathic pain (*p* = 0.009). The use of sleep medication also differed significantly (*p* = 0.016); male participants with mucosal/cutaneous pain reported higher medication use than those with periodontal (*p* = 0.003) and impacted tooth-related pain (*p* = 0.001). No significant differences emerged across pain groups for sleep duration, habitual sleep efficiency, daytime dysfunction, or total PSQI scores (all *p * > 0.05).

Among female participants, pain types differed significantly in several sleep quality parameters (**Supplementary Table 3**). Subjective sleep quality did not vary across groups (*p * > 0.05). Sleep latency differed significantly (*p* = 0.008); women with oncologic pain had higher scores than those with pulpal, periodontal, impacted tooth-related, dental implant-associated, and TMD-related pain, while those with mucosal/cutaneous pain also scored higher than the same groups (all *p * < 0.05). Sleep duration showed significant variation (*p* = 0.001); women with neuropathic pain reported longer sleep duration than those with pulpal, periodontal, and impacted tooth-related pain. Habitual sleep efficiency also differed (*p* = 0.037); women with oncologic pain scored higher than those with pulpal, periodontal, impacted tooth-related, dental implant-associated, and neuropathic pain. Sleep disturbance scores varied significantly (*p* = 0.003); oncologic pain patients scored higher than those with impacted tooth-related and TMD-related pain. The use of sleep medication also differed (*p* = 0.001); women with oncologic pain reported greater use than those with impacted tooth-related and dental implant-associated pain, while those with mucosal/cutaneous pain reported higher use than the same groups. Daytime dysfunction differed significantly (*p* = 0.011); women with mucosal/cutaneous pain had higher scores than those with dental implant-associated and oncologic pain, and those with impacted tooth-related pain scored higher than the same two groups. Finally, total PSQI scores varied significantly (*p* = 0.001); women with oncologic pain scored higher than those with pulpal, impacted tooth-related, and dental implant-associated pain, while those with mucosal/cutaneous pain also scored higher than the same groups. No other significant differences were detected across groups (*p * > 0.05).

## 4. Discussion

This study aimed to comparatively evaluate the distinctive effects of eight different types of orofacial pain on sleep quality, as well as the impact of demographic variables, such as age and gender, on specific sleep parameters.

Shaefer *et al*. [17] reported that nearly all subtypes of orofacial pain have a higher prevalence among females, who tend to have lower pain thresholds and exhibit higher levels of pain perception and expression. In particular, TMD-related pain, neuropathic pain, and burning mouth syndrome—which is classified under mucosal pain—have been noted to occur more frequently and with greater severity in women.

This gender disparity has been attributed to a combination of hormonal fluctuations, the influence of estrogen and progesterone, genetic predispositions, and psychosomatic factors. Additionally, women are reported to have a greater tendency toward pain catastrophizing, which may also increase psychological vulnerability to sleep disturbances [17]. Similarly, Wieckiewicz *et al*. [18] suggested that due to emotional and hormonal differences, women may be more sensitive to changes in sleep patterns, which in turn can create a stronger interaction between pain threshold and sleep quality. The findings of our study are largely consistent with the existing literature. Female participants exhibited significantly longer sleep latency, higher scores in sleep disturbances and daytime dysfunction, and overall higher PSQI scores compared with males. Notably, women in the mucosal and neuropathic pain groups had higher sleep disturbance scores than their male counterparts. These results indicate that gender is a critical factor to consider in sleep quality impairments associated with orofacial pain.

Fiedler *et al*. [19] reported that with increasing age, pain-related sleep fragmentation becomes more frequent, potentially creating a cyclical mechanism that adversely affects both pain perception and sleep integrity. Similarly, Orzeszek *et al*. [20] demonstrated that age has a significant impact on both pain perception and sleep quality in individuals with TMDs. Their findings revealed that older adults experienced more pronounced difficulties with sleep initiation, increased sleep duration, and greater impairment in daytime functioning [20]. Consistent with these findings, age showed significant associations with several PSQI components. As age increased, scores for sleep duration, sleep disturbances, and use of sleep medication also increased, while subjective sleep quality scores decreased. These results suggest that older individuals tend to sleep longer but perceive their sleep as less restful and of lower quality. This supports the notion that, much like gender, age is a critical demographic variable that influences not only the experience of pain but also sleep quality. It may therefore act as a determinant in the interaction between orofacial pain and sleep physiology. In addition, the acute or chronic nature of pain was found to have differential effects on sleep quality. In individuals with chronic pain, sleep latency and sleep disturbance scores were significantly higher, indicating that sleep quality is more persistently impaired in this group. In contrast, acute pain was associated with shorter-term and transient sleep disturbances. These findings highlight the importance of considering pain duration and course when evaluating its impact on sleep quality.

Although the number of studies focusing on the characteristic features of pain associated with oral mucosal lesions remains limited in the literature, recent research has begun to explore their impact on sleep quality. In a study by Abdalla-Aslan *et al*. [21], which included 63 patients diagnosed with acute ulcers, herpes infections, or immune-mediated chronic mucosal diseases (*e.g.*, oral lichen planus, pemphigus vulgaris), approximately 44.4% of participants reported awakening from sleep due to pain. These mucosal pains, often described as “burning” in nature, were associated with high levels of unpleasantness and were linked to sleep fragmentation independently of pain intensity. Furthermore, the frequency of nighttime awakenings was significantly associated with female gender. The authors emphasized that mucosal pain shares characteristics with visceral pain and should be evaluated differently from classical cutaneous pain classifications [21]. In line with these findings, our study revealed that individuals experiencing mucosal/cutaneous pain showed statistically significant deterioration in specific PSQI subcomponents, particularly sleep duration, sleep disturbances, and daytime dysfunction. These results suggest that inflammation of the mucosal surfaces and/or lesions with neuropathic characteristics not only cause physical discomfort, but also significantly impair sleep quality. When interpreted alongside existing literature, our findings support the conclusion that both the sensory and affective components of pain in patients with mucosal/cutaneous involvement should be considered in clinical management, and that sleep quality should be systematically assessed in this population. In clinical practice, patients presenting with mucosal or cutaneous pain should, therefore, be considered a high-risk group for sleep disturbances, and routine screening for sleep quality impairment may facilitate earlier intervention and improved outcomes.

Lavigne and Sessle reported that disrupted sleep patterns are frequently observed in individuals with trigeminal neuralgia and other trigeminal neuropathic pain conditions, with approximately 20% of patients experiencing nighttime awakening episodes [4]. The chronic nature of neuropathic pain can interfere with the sleep-wake cycle, potentially creating a cyclical model in which both pain and insomnia are perpetuated. Mechanisms such as central sensitization, enhanced synaptic transmission, and neuroinflammation—commonly observed in this type of pain—hinder the natural suppression of pain perception during sleep, leading to non-restorative sleep. Some researches further highlighted the role of ectopic discharges, glial cell activation, and dysfunction in inhibitory neural pathways following trigeminal nerve injury as key pathophysiological mechanisms underlying neuralgic pain. These processes are also thought to have a direct impact on sleep regulation [22].

In our study, participants with neuropathic pain demonstrated statistically significant differences in sleep latency, sleep duration, and sleep disturbance scores compared with other pain groups. Notably, increased sleep latency and elevated sleep disturbance scores suggest that these individuals experience difficulties in initiating sleep and maintaining sleep integrity. Interestingly, the relatively longer sleep duration observed in this group may indicate that heightened pain sensitivity, when combined with fatigue, leads to an increased need for sleep. These findings underscore that neuropathic pain produces not only sensory disruptions, but also multifaceted systemic consequences that affect sleep physiology. As such, clinical management of this patient population should incorporate not only pain relief strategies, but also targeted interventions aimed at improving sleep quality. Neuropathic pain is frequently associated with central sensitization, heightened excitability of nociceptive pathways, and impaired inhibitory mechanisms, which collectively diminish the natural downregulation of pain perception during sleep [23, 24]. In addition, neuroinflammatory processes and abnormal ectopic discharges from trigeminal pathways may disrupt sleep-wake regulation and contribute to non-restorative sleep [25, 26]. Similarly, mucosal pain often involves persistent inflammatory activity and lesions with neuropathic features, such as burning mouth syndrome, which can further compromise sleep regulation [23]. These mechanisms may generate continuous nociceptive input and increased unpleasantness that not only interfere with sleep initiation and maintenance, but also raise the likelihood of nighttime awakenings. Together, these processes provide a plausible explanation for the particularly detrimental impact of neuropathic and mucosal/cutaneous pain on sleep quality observed in our study. Moreover, systematic screening for sleep disturbances in patients with neuropathic pain is warranted, as early recognition of impaired sleep may enable timely and multidisciplinary interventions.

Studies specifically addressing the impact of oncologic pain on sleep quality remain limited in the literature, with most studies focusing on overall sleep quality rather than detailed sleep parameters. In female cancer patients, increased sleep latency and nighttime awakenings have been reported, while other studies have emphasized that sleep disturbances may be influenced not only by pain, but also by comorbid factors, such as anxiety and depression [27, 28]. In two separate studies by Nunes *et al*. [29, 30], cancer-related pain was found to interfere with nighttime sleep, and overall sleep quality in these patients was shown to be modulated by tumor type, treatment protocols, and emotional status. Recent prospective longitudinal research in pediatric oncology has demonstrated that pain severity is significantly associated with sleep problems in children undergoing treatment for acute lymphoblastic leukemia [31]. Our study contributes original data to this limited body of literature by examining the effect of oncologic pain on sleep quality in greater detail through PSQI subcomponents. According to our findings, individuals experiencing oncologic pain exhibited significantly higher scores in sleep latency and sleep disturbances, alongside lower scores in sleep duration and habitual sleep efficiency. Moreover, the elevated use of sleep medication in this group suggests that both the physiological burden of pain and the emotional stress associated with the cancer treatment process negatively affect sleep patterns. These results highlight the necessity of incorporating multidisciplinary strategies into the treatment plans of oncologic patients, not only to manage pain effectively but also to preserve and improve sleep quality throughout the course of care. Given the vulnerability of oncologic patients, systematic sleep disturbance screening should be integrated into routine care pathways to ensure timely management of both pain and sleep-related problems.

In a systematic review conducted by Dreweck *et al*. [32], it was reported that patients with painful TMD experience prolonged sleep latency, reduced sleep duration, and overall deterioration in sleep quality. Similarly, in their study, Lavigne and Sessle emphasized that TMD is associated with irregularities in both rapid eye movement (REM) and non–rapid eye movement (non-REM) sleep stages, which may exacerbate pain intensity and result in a bidirectional relationship between sleep disturbances and pain [4]. Furthermore, a 2022 systematic review by Romero *et al*. [33] noted that sleep disorders and poor sleep quality are frequently observed in individuals with temporomandibular joint osteoarthritis (TMJ-OA). Insomnia and sleep apnea were reported as the most prevalent sleep problems in TMD patients [33]. These studies suggest that factors such as bruxism, parafunctional habits, and sleep fragmentation may contribute to the exacerbation of TMD symptomatology. Our study adds to this body of evidence by offering a detailed analysis of the impact of TMD-related pain on PSQI subcomponents. According to our findings, patients with TMD demonstrated higher sleep latency and sleep disturbance scores compared with many other pain groups, along with a significantly elevated total PSQI score. Moreover, the significantly increased daytime dysfunction scores in this group suggest that poor nighttime sleep quality adversely affects daytime performance and overall functioning. It is hypothesized that parafunctional activities occurring during sleep—such as bruxism—and increased masticatory muscle activity may aggravate TMD symptoms and contribute to sleep fragmentation. Based on these findings, it becomes evident that comprehensive management of TMD should not be limited to pain control alone, but must also include a thorough evaluation and improvement of sleep quality.

Studies that specifically examine the effects of pulpal and periodontal pain on sleep quality are notably scarce in the literature. Most investigations tend to group these pain types under the general category of “dental pain”, without adequately addressing the distinct impacts of each type on sleep physiology [12, 19]. This lack of specificity limits the ability to distinguish how different dental pain origins influence sleep patterns. In our study, pulpal and periodontal pain were analyzed as separate entities, allowing for a more precise understanding of their effects on sleep quality. Our findings revealed that individuals with pulpal pain had significantly shorter sleep durations. Additionally, higher scores in sleep disturbances and daytime dysfunction in this group indicate disruption in sleep continuity. Among those with periodontal pain, elevated scores in sleep disturbances and daytime dysfunction were also observed. Furthermore, total PSQI scores in this group were significantly higher when compared with individuals with pain related to impacted teeth or dental implants. These results suggest that pulpal and periodontal pain are not merely localized inflammatory conditions, but may act as systemic stressors that adversely affect both sleep regulation and overall quality of life.

The number of studies directly evaluating the effects of pain associated with impacted teeth on sleep quality is quite limited. Addressing this gap, our study conducted a detailed analysis of sleep quality parameters in individuals with impacted tooth-related pain. According to our results, this group exhibited shorter sleep durations compared with several other pain groups. Additionally, elevated daytime dysfunction scores may reflect fatigue and concentration difficulties caused by sleep interruptions during the night. Impacted tooth pain is often described as throbbing or sharp in nature and tends to intensify during nighttime hours. This characteristic can hinder both sleep initiation and maintenance. The findings of our study indicate that pain associated with impacted teeth should not be regarded solely as a localized oral issue, but rather as a condition with broader implications for general health and quality of life.

Although few studies have directly examined the relationship between pain associated with dental implants and sleep quality, some reports have highlighted the adverse effects of post-implant pain on quality of life, particularly sleep. Lobbezoo *et al*. [7] noted that pain following dental procedures, especially implant treatments, may lead to nighttime awakenings, sleep fragmentation, and reduced sleep duration. This was attributed to persistent sensitivity in the implant region and discomfort throughout the night, both of which may impair sleep initiation and maintenance. In our study, we observed similar trends; individuals experiencing implant-related pain demonstrated poorer sleep quality parameters. Specifically, these individuals showed lower sleep duration and habitual sleep efficiency scores compared with other pain groups. In addition, their sleep disturbance and sleep medication use scores were relatively elevated. These findings indicate that implant-related pain is a significant disruptor of sleep quality and emphasize the need for postoperative pain management strategies that address not only physical discomfort, but also sleep hygiene.

Subjective sleep quality reflects an individual’s perception of their sleep experience and is considered an important indicator of pain-related sleep disruption. Recent studies show that sleep problems associated with pain are more frequently observed among women, who tend to rate their sleep less favorably [9]. Similarly, individuals with chronic pain—particularly women—often report poorer subjective sleep quality, findings that have been linked to depressive symptoms and anxiety [34]. These patterns suggest that psychological factors accompanying pain may significantly modulate sleep perception. In our study, female participants had significantly higher subjective sleep quality scores than males, supporting the notion that women may experience pain with more intense emotional components. Furthermore, our study makes a contribution to the literature by analyzing subjective sleep quality across different types of orofacial pain. According to our results, individuals with neuropathic and mucosal/cutaneous pain reported significantly lower subjective sleep quality scores than other pain groups. This is likely due to the sharp, burning, and persistent discomfort often associated with these pain types, which directly compromises the sleep experience.

Habitual sleep efficiency—defined as the proportion of time spent in bed that is actually spent sleeping—is a critical measure of sleep productivity. Although specific data on this parameter are limited in the literature, Matsuka *et al*. [35] reported that individuals with chronic orofacial pain tend to experience reduced overall sleep quality and disrupted sleep patterns, though they did not present quantitative findings on habitual sleep efficiency. Ekici found that among patients with TMD, increased pain severity was associated with poorer sleep efficiency, as measured by PSQI subcomponents [36]. Our study contributes to the literature by assessing habitual sleep efficiency across a range of orofacial pain types—not only TMD. Particularly in individuals with oncologic and mucosal/cutaneous pain, habitual sleep efficiency scores were found to be significantly lower. These findings suggest that the chronic and persistent nature of pain in these groups severely impairs the restorative capacity of sleep.

Pharmacological interventions are frequently employed to manage sleep disturbances in individuals with chronic pain. Su *et al*. [37] reported that sleep medication use is common among patients with chronic orofacial pain, but also highlighted potential risks associated with long-term use, including tolerance, dependence, and cognitive side effects. Ekici similarly noted that sleep medication scores increase in patients with TMD as pain severity rises [36]. In our study, this parameter was assessed not only in TMD, but also across a broad range of orofacial pain types. Notably, individuals with mucosal/cutaneous and oncologic pain demonstrated significantly higher levels of sleep medication use. In addition, female participants were found to use sleep medications more frequently than males. These findings reflect differing tendencies toward pharmacological coping based on pain type and gender, underscoring the need for a multidisciplinary, holistic approach to managing sleep disorders—rather than relying solely on medication-based strategies.

The main strength of this study lies in its systematic comparison of eight different orofacial pain types in relation to sleep quality, an approach rarely addressed in the literature. The inclusion of demographic factors and clinical pain characteristics—including location, intensity, duration, frequency, and acute versus chronic status—adds further clinical depth. However, several limitations should be acknowledged. The cross-sectional design precludes causal inference, and reliance on the PSQI without objective measures, such as polysomnography, may have overlooked certain physiological aspects of sleep. In addition, the absence of multiple testing corrections or multivariable analyses may increase the risk of type I error. Another important limitation is the unequal distribution of sample sizes across pain groups. Some subgroups, particularly neuropathic and oncologic pain, included relatively few patients, which reduced statistical power, widened confidence intervals, and limited the generalizability of subgroup findings. These results should therefore be regarded as exploratory and hypothesis-generating. Finally, the single-center, region-specific setting further constrains external validity. Future research with larger, multi-center cohorts and objective sleep assessments will be essential to confirm and extend these findings.

## 5. Conclusions

This study demonstrates that different types of orofacial pain have distinct and measurable impacts on sleep quality. Notably, neuropathic and mucosal/cutaneous pain were associated with the most severe impairments, in terms of total PSQI scores and multiple subcomponents. Female participants exhibited lower overall sleep quality compared with males, and age was found to influence specific sleep parameters. These findings suggest that not only the presence of pain, but also the type of pain and individual demographic factors significantly influence sleep processes. Whereas most previous studies have focused on a single type of orofacial pain, the present study offers a novel contribution by comparatively analyzing a broad spectrum of eight orofacial pain types within the same sample. By detailing the relationship between these pain types and specific sleep components, the study underscores the necessity of adopting a sleep-conscious approach to pain management in dental clinical practice. The data indicate that in dental settings, the evaluation of sleep disturbances accompanying pain should be conducted systematically, rather than focusing solely on pain control. Recognizing sleep disruptions specific to each pain type and tailoring treatment strategies accordingly may improve both patient satisfaction and treatment outcomes. In this regard, a multidisciplinary approach would provide a more effective framework for addressing both the physiological and quality-of-life dimensions of orofacial pain management.

Beyond raising awareness of which patients are at higher risk of sleep disturbance, our findings also provide practical guidance for clinicians. Patients with orofacial pain types associated with chronic sleep impairment could be considered for preventive referral to a sleep specialist. In addition, clinicians may recommend simple, evidence-based strategies—such as advising patients to take prescribed analgesics before bedtime, offering education on sleep hygiene, and encouraging non-pharmacological approaches (*e.g.*, structured sleep methods, evidence-based online resources). Incorporating these measures into routine dental practice may help mitigate the broader impact of orofacial pain on sleep quality and overall well-being.

## Supplementary Material

Supplementary material associated with this article can be found, in the online version, at https://files.jofph.com/ files/article/2010589184798932992/attachment/ Supplementary%20material.docx.


