# Supplementary material for: Type-specific effects of orofacial pain on sleep quality: a cross-sectional clinical study
Source: J Oral Facial Pain Headache. 2026 Jan 12;40(1):127–37. doi: 10.22514/jofph.2026.011 (PMC12853164; doi:10.22514/jofph.2026.011)
Supplement: Supplementary file 1 [file Supplementary-material.docx]

****Supplementary materials****

Supplementary Table 1. Simplified analogies for clinical interpretation of pain types.

| Pain Type | Common Clinical Analogy (for easier interpretation) |
| --- | --- |
| Pulpal pain | Pain leading to root canal treatment (irreversible pulpitis) |
| Periodontal pain | Pain managed with scaling and root planing (periodontitis-related) |
| Impacted tooth pain | Pain from pericoronitis around impacted mandibular third molars |
| Dental implant pain | Peri-implantitis/post-implant discomfort |
| TMD-related pain | Temporomandibular joint or muscle pain during function |
| Mucosal/cutaneous pain | Burning mouth syndrome, oral ulcers, lichen planus |
| Neuropathic pain | Trigeminal neuralgia-related sharp, electric-like pain |
| Oncologic pain | Post-radiation or malignancy-associated pain |

**TMD: temporomandibular disorders.**

Supplementary Table 2. PSQI subcomponents and total score by pain type in male participants.

| PSQI Component | Pulpal Pain | Periodontal Pain | Impacted tooth-related pain | Dental implant pain | TMD-related Pain | Mucosal/cutaneous Pain | Neuropathic Pain | Oncologic Pain |
| --- | --- | --- | --- | --- | --- | --- | --- | --- |
| Subjective Sleep Quality | 0.27 ± 0.58; 0 (0–0.5) | 0.56 ± 0.76; 0 (0–1) | 0.14 ± 0.36; 0.0 (0–0) | 0.36 ± 0.81; 0 (0–1) | 0.00 ± 0.00; 0 (0–0) | 0.00 ± 0.00; 0 (0–0) | 0.00 ± 0.00; 0 (0–0) | 1.00 ± 1.33; 0.0 (0–2) |
| Sleep Latency | 1.23 ± 0.77; 1 (1.0–2.0) | 0.92 ± 0.90; 1 (0–2) | 1.00 ± 0.96; 1.0 (0–2) | 1.00 ± 0.63; 1 (0–2) | 0.60 ± 0.52; 1 (0–1) | 1.67 ± 0.52; 2 (1–2) | 2.00 ± 0.00; 2 (2–2) | 1.00 ± 1.15; 1.0 (0–2) |
| Sleep Duration | 0.67 ± 0.88; 0 (0.0–1.0) | 0.60 ± 1.14; 0 (0–1) | 0.14 ± 0.36; 0.0 (0–0) | 0.55 ± 1.21; 0 (0–1) | 0.60 ± 0.84; 0 (0–1) | 0.00 ± 0.00; 0 (0–0) | 0.00 ± 0.00; 0 (0–0) | 0.00 ± 0.00; 0.0 (0–0) |
| Habitual Sleep Efficiency | 0.40 ± 0.72; 0 (0.0–1.0) | 0.12 ± 0.33; 0 (0–0) | 0.07 ± 0.27; 0.0 (0–0) | 0.00 ± 0.00; 0 (0–0) | 0.10 ± 0.32; 0 (0–0) | 0.00 ± 0.00; 0 (0–0) | 0.00 ± 0.00; 0 (0–0) | 0.10 ± 0.32; 0.0 (0–0) |
| Sleep Disturbances | 1.13 ± 0.35; 1 (1.0–1.0) | 1.16 ± 0.55; 1 (1–2) | 1.00 ± 0.55; 1.0 (1–1) | 1.18 ± 0.40; 1 (1–1) | 1.00 ± 0.00; 1 (1–1) | 2.00 ± 0.00; 2 (2–2) | 1.00 ± 0.00; 1 (1–1) | 1.40 ± 0.52; 1.0 (1–2) |
| Use of Sleep Medication | 1.00 ± 0.37; 1 (1.0–1.0) | 0.92 ± 0.70; 1 (0–1) | 0.71 ± 0.47; 1.0 (0–1) | 1.00 ± 0.63; 1 (0–1) | 1.00 ± 0.00; 1 (1–1) | 1.67 ± 0.52; 2 (1–2) | 1.00 ± 0.00; 1 (1–1) | 1.40 ± 0.52; 1.0 (1–2) |
| Daytime Dysfunction | 0.40 ± 0.62; 0 (0.0–1.0) | 0.40 ± 0.57; 0 (0–1) | 0.00 ± 0.00; 0.0 (0–0) | 0.73 ± 0.90; 0 (0–1) | 0.40 ± 0.52; 0 (0–1) | 0.33 ± 0.52; 0 (0–1) | 0.00 ± 0.00; 0 (0–0) | 0.40 ± 0.52; 0.0 (0–1) |
| Total PSQI Score | 5.10 ± 2.04; 5 (4.0–6.0) | 4.68 ± 2.37; 5 (3–6) | 3.07 ± 1.49; 3.5 (2–4) | 4.82 ± 1.94; 5 (4–6) | 3.70 ± 1.25; 4 (3–4) | 5.67 ± 1.37; 6 (5–7) | 4.00 ± 0.00; 4 (4–4) | 5.30 ± 3.30; 3.5 (3–7) |

Data are presented as Mean ± SD; Median (Interquartile Range). Kruskal-Wallis test. *p* < 0.05 indicates statistical significance. PSQI: Pittsburgh Sleep Quality Index; TMD: temporomandibular disorders.

Supplementary Table 3. PSQI subcomponents and total score by pain type in female participants.

| PSQI Component | Pulpal Pain | Periodontal Pain | Impacted tooth-related pain | Dental implant pain | TMD-related Pain | Mucosal/cutaneous Pain | Neuropathic Pain | Oncologic Pain |
| --- | --- | --- | --- | --- | --- | --- | --- | --- |
| Subjective Sleep Quality | 0.83 ± 0.93; 0 (0–1) | 0.83 ± 0.99; 0 (0–1) | 0.80 ± 1.14; 0.0 (0–1) | 0.00 ± 0.00; 0 (0–0) | 0.89 ± 1.03; 1 (0–1) | 1.14 ± 1.03; 1 (0–2) | 0.80 ± 1.03; 0 (0–1) | 0.00 ± 0.00; 0 (0–0) |
| Sleep Latency | 1.36 ± 0.93; 1 (1–2) | 1.45 ± 0.78; 1 (1–2) | 1.24 ± 0.87; 2.0 (1–2) | 1.22 ± 0.83; 1 (1–2) | 1.32 ± 1.09; 1 (0–2) | 2.14 ± 0.86; 2 (2–3) | 2.00 ± 1.15; 2 (1–3) | 2.50 ± 0.58; 2.5 (2–3) |
| Sleep Duration | 0.26 ± 0.73; 0 (0–0) | 0.38 ± 0.77; 0 (0–1) | 0.20 ± 0.49; 0.0 (0–0) | 0.67 ± 0.50; 1 (0–1) | 0.53 ± 0.76; 0 (0–1) | 0.86 ± 1.17; 0 (0–1) | 1.00 ± 0.67; 1 (1–1) | 1.00 ± 1.15; 1.0 (0–2) |
| Habitual Sleep Efficiency | 0.20 ± 0.51; 0 (0–0) | 0.21 ± 0.49; 0 (0–0) | 0.26 ± 0.66; 0.0 (0–0) | 0.00 ± 0.00; 0 (0–0) | 0.32 ± 0.57; 0 (0–1) | 0.43 ± 0.51; 0 (0–1) | 0.00 ± 0.00; 0 (0–0) | 1.50 ± 1.73; 1.5 (0–3) |
| Sleep Disturbances | 1.43 ± 0.54; 1 (1–2) | 1.41 ± 0.56; 1 (1–2) | 1.28 ± 0.73; 1.0 (1–2) | 1.56 ± 0.53; 2 (1–2) | 1.21 ± 0.70; 1 (1–2) | 1.57 ± 0.51; 2 (1–2) | 1.80 ± 0.42; 2 (2–2) | 2.50 ± 0.58; 2.5 (2–3) |
| Use of Sleep Medication | 1.10 ± 0.61; 1 (1–1) | 1.21 ± 0.67; 1 (1–1) | 0.96 ± 0.67; 1.0 (0–1) | 0.56 ± 0.53; 1 (0–1) | 1.26 ± 0.64; 1 (1–2) | 1.71 ± 0.73; 2 (1–2) | 1.60 ± 0.84; 1 (1–2) | 2.00 ± 0.00; 2.0 (2–2) |
| Daytime Dysfunction | 0.52 ± 0.77; 0 (0–1) | 0.66 ± 0.81; 0 (0–1) | 0.72 ± 0.78; 1.0 (0–1) | 0.00 ± 0.00; 0 (0–0) | 0.47 ± 0.69; 0 (0–1) | 1.00 ± 0.78; 1 (0–1) | 0.40 ± 0.52; 0 (0–1) | 0.00 ± 0.00; 0.0 (0–0) |
| Total PSQI Score | 5.70 ± 2.91; 5 (4–7) | 6.14 ± 2.79; 6 (5–8) | 5.46 ± 3.02; 4.5 (4–7) | 4.00 ± 0.71; 4 (4–4) | 6.00 ± 2.67; 5 (4–7) | 8.86 ± 3.66; 9 (7–11) | 7.60 ± 3.03; 8 (6–9) | 9.50 ± 2.89; 9.5 (8–11) |

Data are presented as Mean ± SD; Median (Interquartile Range). Kruskal-Wallis test. *p* < 0.05 indicates statistical significance. PSQI: Pittsburgh Sleep Quality Index; TMD: temporomandibular disorders.
